# Surface-Localized Crosslinked MEW PCL–Hydrogel Scaffolds with Tunable Porosity for Enhanced Cell Adhesion and Viability

**DOI:** 10.3390/polym17152086

**Published:** 2025-07-30

**Authors:** Yixin Li, Le Kang, Kai Cao

**Affiliations:** Key Laboratory of Textile Science & Technology of Ministry of Education, College of Textiles, Donghua University, Shanghai 201620, China; lyx2862379848@163.com (Y.L.); lekang@mail.dhu.edu.cn (L.K.)

**Keywords:** surface-confined crosslinking, melt electrowriting, hydrogel, porous composite scaffold

## Abstract

Hydrogel is widely used as a scaffolding material for tissue engineering due to its excellent cytocompatibility and potential for biofunctionalization. However, its poor mechanical property limits its further application. Fabrication of fiber-reinforced hydrogel composite scaffolds has emerged as a solution to overcome this problem. However, existing strategies usually produce nonporous composite scaffolds, where the interfiber pores are completely filled with hydrogel. This design can hinder oxygen and nutrient exchange between seeded cells and the culture medium, thereby limiting cell invasion and colonization within the scaffold. In this study, sodium alginate (SA) hydrogel was exclusively grafted onto the surface of the constituent fibers of the melt electrowritten scaffold while preserving the porous structure. The grafted hydrogel amount and pore size were precisely controlled by adjusting the SA concentration and the crosslinking ratio (SA: CaCl_2_). Experimental results demonstrated that the porous composite scaffolds exhibited superior swelling capacity, degradation ratio, mechanical properties, and biocompatibility. Notably, at an SA concentration of 0.5% and a crosslinking ratio of 2:1, the porous composite scaffold achieved optimal cell adhesion and viability. This study highlights the critical importance of preserving porous structures in composite scaffolds for tissue-engineering applications.

## 1. Introduction

Tissue engineering (TE) has emerged as a highly promising paradigm for the regeneration of damaged or diseased tissues, offering potential solutions where natural healing processes are insufficient [1]. Central to the success of TE strategies is the design and fabrication of scaffolds that faithfully recapitulate the intricate physiological characteristics [2] and hierarchical structure of native extracellular matrix (ECM) [3]. Achieving this biomimicry necessitates the processing of appropriate biomaterials through sophisticated methods. Established methods such as freeze-drying [4], gas foaming [5], electrospinning [6], and additive manufacturing (3D printing) have been extensively explored [7], each offering distinct advantages and limitations in scaffold architecture and functionality.

Among these evolving technologies, melt electrowriting (MEW) has rapidly ascended as a competitive frontrunner. MEW uniquely converges the precision of additive manufacturing with the thin-fiber-producing capabilities of electrospinning [8], offering significant advantages: a solvent-free process eliminates cytotoxic solvent residues, the production of fibers with diameters comparable to cells (sub-10 µm range) provides topographical cues [9], and unparalleled microarchitectural customizability enables the precise engineering of pore size, geometry, and fiber orientation [10]. In recent years, the library of biomaterials amenable to MEW processing has expanded considerably [11], encompassing polymers like Poly(lactic-co-glycolic acid) (PLGA [12]), Poly(L-lactic acid) (PLLA [13]), and Poly(vinylidene difluoride) (PVDF) [14]. Nevertheless, polycaprolactone (PCL) persists as the ‘gold-standard’ material for MEW [15,16,17]. Its dominance stems from exceptional printability, well-established biocompatibility, and a relatively low melting point (~60 °C) [18,19]. However, the inherent hydrophobicity of PCL presents a critical limitation [20]: it fails to provide an optimally favorable microenvironment for cell adhesion [21], spreading, and proliferation [22]. Furthermore, PCL’s intrinsic lack of bioactive moieties and the challenges associated with its effective biofunctionalization significantly hinder its broader application [23], particularly in demanding contexts like cartilage regeneration which requires robust cell–matrix interactions and specific biochemical signaling.

In stark contrast, hydrogels—three-dimensional, hydrophilic polymer networks crosslinked either physically or chemically—excel in creating cell-friendly environments [24]. Their high water content (mimicking the hydrated nature of native ECM), inherent hydrophilicity, tunable soft mechanical properties (approximating many soft tissues [25]), and ease of incorporation of bioactive molecules (e.g., peptides, growth factors) collectively foster excellent cell affinity, adhesion, proliferation [26], and phenotype maintenance [27]. Natural hydrogels, prized for their biocompatibility and biodegradability, are frequently employed in cartilage tissue engineering. Typical examples include sodium alginate [28] (SA), hyaluronic acid (HA [29]), agarose, chitosan (CS [30]), gelatin [31], and collagen [32]. SA and CS, in particular, have garnered significant attention for cartilage repair applications due to their non-toxicity, excellent biocompatibility, and controllable degradation profiles [33]. Illustrative work includes that of Man et al., who fabricated composite scaffolds using CS hydrogel combined with demineralized bone matrix for rabbit cartilage repair [34], and Yuan et al., who developed a mechanically reinforced dual-network hydrogel scaffold based on bovine serum albumin (BSA), sodium alginate, and hydroxyapatite nanowires (B-S-H) [35].

However, a fundamental challenge persists with many natural hydrogels, including SA and CS: their frequently inadequate mechanical properties, especially under dynamic loading conditions typical of articular joints [36]. This mechanical deficiency often compromises their utility as standalone scaffolds for load-bearing tissue repair [37]. To circumvent this limitation, researchers have pursued the strategy of combining MEW-produced fibrous structures with hydrogels, creating fiber-reinforced hydrogel composites [38]. The rationale is to leverage the mechanical robustness and structural integrity of the MEW scaffold to reinforce the otherwise weak hydrogel matrix, while the hydrogel component provides the desired bioactivity and cell-instructive properties. For instance, Qiao et al. successfully embedded PCL melt-electrospun scaffolds within a network of gelatin methacryloyl (GelMA) and polyethylene glycol (PEG), yielding an ‘amber-like’ bio-scaffold capable of effectively inducing mesenchymal stem cell (MSC) chondrogenic differentiation [39]. While such composites show promise, a critical drawback emerges: the hydrogel typically completely infiltrates and fills the interstitial pores between the electrospun fibers. This pore occlusion creates a significant barrier to the essential diffusion of oxygen, nutrients, and metabolic waste products. Consequently, this architectural limitation can severely impede deep cell migration, uniform cell distribution, long-term viability within the scaffold core, and ultimately, the formation of homogeneous neo-tissue.

To directly address this pivotal challenge of compromised mass transport in conventional fiber-reinforced hydrogels, a modified fabrication strategy is proposed herein. This approach focuses on grafting the hydrogel component exclusively onto the surface of the MEW-derived fibers, while meticulously preserving the interconnected macro- and micro-pores within the fibrous network. This surface-localized hydrogel coating aims to provide the necessary biointerface for enhanced cell–material interactions without sacrificing the vital porosity required for unimpeded diffusion. Furthermore, the thickness and properties of this surface-bound hydrogel layer can be precisely modulated by varying the degree of crosslinking. In this study, these porous composite scaffolds were systematically investigated in terms of their morphology, chemical composition, mechanical properties, and, crucially, performance in supporting in vitro chondrogenesis and cartilage-like matrix deposition. Rigorous comparisons are made against relevant controls: pristine MEW fiber scaffolds, bulk hydrogel scaffolds, and conventional composite scaffolds where the hydrogel fully occupies the pores. It is assumed that this surface-grafting strategy constitutes a significant advancement, offering a novel pathway to engineer fiber–hydrogel composite scaffolds that synergistically enhance cell adhesion, viability, migration, and functional tissue formation by maintaining an open, diffusion-permissive architecture alongside a bioactive fiber surface.

## 2. Materials and Methods

### 2.1. Materials

PCL with Mw 50,000 g/mol, Perstrop Capa 6500 ( CAS: 24980-41-4, KXL Plastic Raw Material Co., Ltd., Dongguan, China), SA (CAS:9005-38-3, Aladdin, Shanghai, China), CaCl_2_ (CAS:10043-52-4, Sinopharm Group, Shanghai, China), NaOH (CAS:1310-73-2, Sinopharm Group, Shanghai, China), sodium dodecyl sulfate (CAS: 151-21-3, Sinopharm Group, Shanghai, China) and CS (CAS: 9012-76-4, Sinopharm Group, Shanghai, China) with degree of deacetylation 80–95%, acetic acid ( CAS: 64-19-7, Titan, Shanghai, China), PBS (Solarbio, Beijing, China).

### 2.2. MEW Device

**Figure 1:**
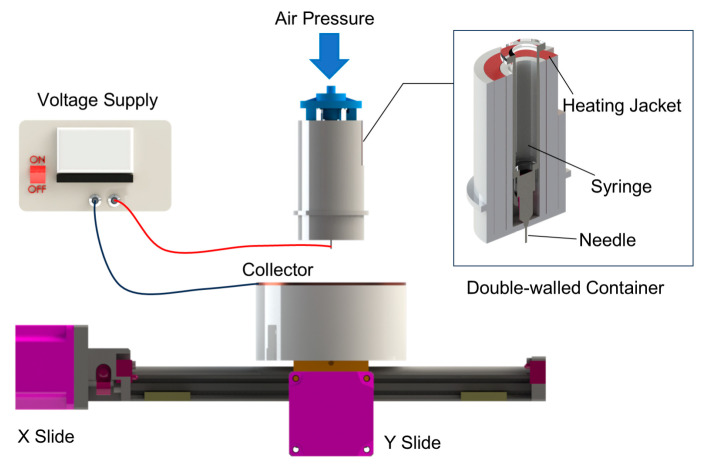
Melt electrowriting (MEW) device used in this study.

Figure 1 displays the self-designed melt electrowriting (MEW) device. A ceramic syringe is positioned within a tubular, double-walled ceramic container, sourced from Guangdong Blue Whale Ceramic Technology Co., Ltd, Dongguan, China. This double-walled container is mounted on a Z-axis slide, enabling its vertical movement and the distance between the needle and the collector. A heating jacket (purchased from Jiangsu Xiaochuangxin Thermal Energy Technology Co., Ltd., Wuxi, China) is placed between the outer and inner walls of the container. At the tip of the syringe, a 24G needle is connected to a high-voltage power supply provided by Dalian Teslaman Technology Co., Ltd. (Dalian, China). A grounded, circular copper plate (15 cm in diameter) serves as the collector, mounted on a translational stage propelled by slides enabling movement in the X and Y directions. The positioning accuracy of the slides is 1 μm. All slides are provided by Suzhou Zhide Automatic Control Co., Ltd., Suzhou, China.

### 2.3. Scaffold Fabrication

Using the customized MEW device, polycaprolactone (PCL) fibrous scaffolds were fabricated. PCL pellets were first loaded into the syringe barrel and heated in an oven at 80 °C for 12 h to achieve a uniform melt state. The PCL-loaded syringe barrel was then kept at room temperature until needed. Before each experiment, the syringe was placed within the heating container’s inner wall and preheated to 90 °C for 30 min. The key parameters of the melt electrowriting (MEW) process were as follows: applied voltage, 3.0 kV; air pressure, 0.05 MPa; needle temperature, 90 °C; needle-to-collector gap, 3 mm; collector translational speed, 7 mm/s; exposed needle length, 2 mm; fiber spacing, 0.5 mm; number of layers, 40; scaffold dimensions, 2 cm × 2 cm; ambient temperature, 24 ± 2 °C; and relative humidity, 35–40%.

#### 2.3.1. Surface Grafting of Hydrogel onto Fibrous Scaffolds

**Figure 2:**
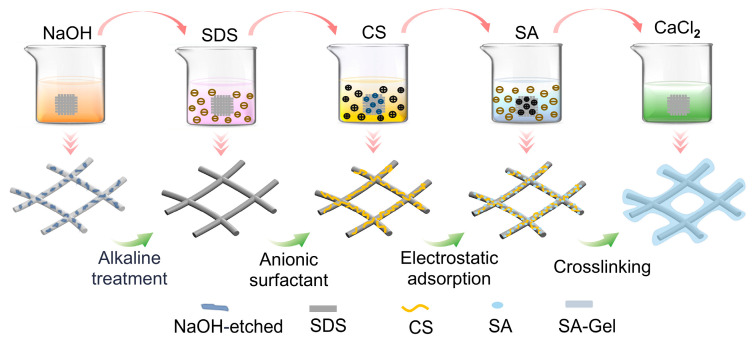
Schematized preparation process of porous composite scaffold.

The preparation process of porous composite scaffolds has been schematized in Figure 2 with details as follows.

(a) Hydrophilic surface modification of the fibrous scaffold: The MEW fibrous scaffolds were immersed in NaOH solution (2 mol/L) and stirred at 100 rpm for 1 h to obtain the alkaline-treated scaffolds. Then the alkaline-treated scaffolds were rinsed with deionized water to achieve a neutral pH, followed by drying at 37 °C for 2 h.
(b) Anionic surfactant SDS treatment: A 5% SDS aqueous solution (20 mL) was prepared. The alkaline-treated scaffolds were immersed in the SDS solution for 20 min, followed by sonication for 30 min, to obtain the SDS-treated scaffold. The SDS-treated scaffold was then rinsed with deionized water and dried at room temperature for 2 h.
(c) Grafting of CS: A 3 wt% CS and 10 wt% acetic acid mixed solution was prepared. The SDS-treated scaffold was then immersed in the CS solution for 4 h, followed by rinsing with ethanol and deionized water to remove any excess CS. Finally, the CS-coated scaffolds were dried at room temperature for 2 days.
(d) Surface grafting of hydrogel: The CS-coated scaffolds were placed into 0.5 wt% and 1 wt% sodium alginate (SA) aqueous solutions for 1 h. Then the SA-coated scaffolds were immersed into CaCl_2_ solutions of different concentrations for rapid crosslinking to obtain the porous composite scaffolds. Specifically, the 0.5 wt% SA-coated scaffolds were immersed into CaCl_2_ solutions of 1, 2, and 4 wt% (denoted as PCL-0.5%SA-2:1, -4:1, and -8:1), while the 1 wt% SA-coated scaffolds were immersed into CaCl_2_ solutions of 2, 4, and 8 wt% (denoted as PCL-1%SA-2:1, -4:1, and -8:1). The six as-prepared samples were freeze-dried for 48 h and stored at room temperature for further use.

#### 2.3.2. Preparation of Control Samples

(a) Nonporous composite scaffold (PCL-SA- NP)

The pristine MEW PCL scaffold was placed into the 1 wt% SA hydrogel precursor solution, and a certain amount of 2 wt% CaCl_2_ solution was added to form the hydrogel, fully encapsulating the scaffold and creating a nonporous structure.

(b) Hydrogel-only scaffold (SA-Gel)

A total of 2 mL 1 wt% SA solution was mixed with an equal volume of 2 wt% CaCl_2_ solution and left for 10 min to form the SA-Gel samples.

### 2.4. Morphology and Composition Analysis

A FLEX1000 scanning electron microscope (SEM, Hitachi Co., Ltd., Tokyo, Japan) was used to observe the pristine MEW fibrous scaffolds. An optical microscope (GMM-580P, Shanghai Guangmi Instrument Co., Ltd., Shanghai, China) was used to observe the porous composite scaffolds. Image Pro Plus was used to determine fiber diameter and spacing.

Fourier transform infrared (FTIR) analysis was conducted on a Spectrum Two spectrometer (Perkin Elmer Co., Ltd., Shelton, CT, USA). The wavelength resolution is 1 cm^−1^ with a 400–4000 cm^−1^ range. X-ray diffraction (XRD) analysis was conducted using a Bruker D8 X-ray diffractometer (Bruker Co., Ltd., Steißlingen, Germany), tested at room temperature with a scan rate of 5°/min and a 2θ range of 5–60°.

### 2.5. Characterization of Pore Features

#### 2.5.1. Pore Size

**Figure 3:**
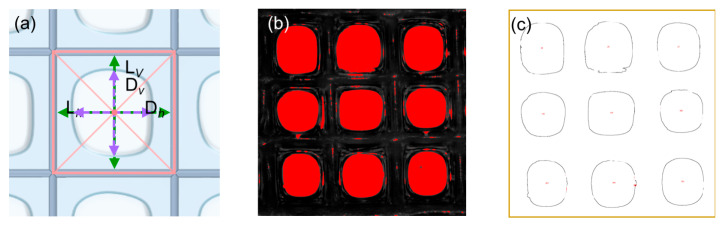
(**a**) Schematic of a porous composite scaffold. A cross was drawn within each cell, connecting the midpoints of the parallel sides. The cross intersects the fibers at four points and the edges of the pore at another four points. These eight points are used to calculate the fiber spacing and pore size in the horizontal and vertical directions, which are *L_h_*, *L_v_*, *D_h_*, and *D_v_*, respectively. (**b**,**c**) illustrate the calculation process for porosity. Specifically, the captured image was binarized and the edges of all closed void regions were identified. The void regions with an area larger than a critical value constitute pores. The porosity is calculated through dividing the number of pixels within the pores by those of the whole image.

The images of composite fiber scaffolds (3 × 3 cells) were imported into Image Pro Plus for pore size analysis. To calculate the pore size, a cross was drawn within each cell, connecting the midpoints of the parallel sides (Figure 3a). The cross intersects the fibers at four points. The distances between the two intersection points along the horizontal and vertical directions were measured and denoted as *L_h_* and *L_v_*, respectively. These distances for all cells were then averaged to obtain the final pore size of the scaffold.

#### 2.5.2. Hydrogel Thickness

The fiber spacings in the horizontal and vertical directions are denoted by *D_h_* and *D_v_*, respectively (Figure 3a). For each cell, the hydrogel thickness in each direction is then calculated as half the difference between the fiber spacing and the corresponding pore size, i.e., (*D_h_* − *L_h_*)/2 horizontally and (*D_v_* − *L_v_*)/2 vertically. The mean of these values across all cells is then reported as the sample’s overall hydrogel thickness.

#### 2.5.3. Porosity

The images of the composite scaffolds (3 × 3 cells) captured under the optical microscope were also imported into Image Pro Plus for further processing. The images were converted to grayscale and binarized to distinguish the pores from other parts. The area of all the void regions was calculated and the porosity was then calculated using the following formula:

(1) $$P=\frac{A_{r}}{A}$$

where *p* is the porosity of the composite scaffold (%), *A_r_* is the area summation of all void regions, and *A* is the total scaffold area, both measured in pixels^2^.

### 2.6. Swelling Behavior

The porous composite scaffolds were cut into small 5 × 5 mm squares, weighed, and then placed into a centrifuge tube containing 20 mL of PBS (pH = 7.4). Then the scaffolds were weighed at intervals of 30 min, with the mass of the scaffolds measured after 30, 60, 90, and 120 min. The swelling ratio was then calculated using the following formula:

(2) $$S=\frac{W_{t}-W_{0}}{W_{0}}$$

where *S* is the swelling ratio of the composite scaffold (%), *W*_0_ the initial mass of the sample, and *W_t_* the mass measured at a specific time point, with the unit in grams (g).

### 2.7. Degradation

The samples prepared for the swelling performance test were weighed and placed into PBS. Every 7 days, the samples were weighed, and this process was repeated over 42 days to observe the degradation behavior of the composite scaffold. The degradation ratio was calculated using the following formula:

(3) $$D=\frac{D_{t}-D_{0}}{D_{0}}$$

where *D* is the degradation ratio of the composite scaffold (%), $D_{0}$ the initial mass of the sample, and $D_{t}$ the mass measured at a specific time point, with the unit in grams (g).

### 2.8. Tensile Testing

The pristine PCL fibers, hydrogel, and composite scaffolds (porous and nonporous) were cut into rectangular strips (10 mm × 3 mm). Tensile strength and elongation at break tests were performed using an XQ-1C tensile testing machine (Shanghai Xinxian Instrument Co., Ltd., Shanghai, China). The stress–strain curves were obtained at a stretching speed of 40 mm/s and a clamping distance of 10 mm.

### 2.9. Cell Culture

Bone marrow mesenchymal stem cells (BMSCs) were isolated from the bone marrow of SD rats (purchased from Cyagen Biosciences, Santa Clara, CA, USA). The cells were cultured in Dulbecco’s Modified Eagle’s Medium/Ham’s F-12 (DMEM/F12) supplemented with 1% penicillin/streptomycin and 10% fetal bovine serum (FBS) (Gibco, Waltham, MA, USA). The cells were maintained in a 37 °C incubator with 5% CO_2_, and the culture medium was replaced every two days. Only passages 3 to 5 were used in this study. Prior to testing, scaffold samples were rinsed with deionized water and phosphate-buffered saline (PBS), followed by sterilization under ultraviolet radiation for 24 h.

### 2.10. Biocompatibility Testing

Different types of scaffold samples were cultured with rabbit bone marrow mesenchymal stem cells in 96-well plates at a cell density of 3000 cells per well, with complete culture medium serving as the blank control. After incubation for 24 and 48 h, AM/PI live/dead staining solution (Beyotime, Shanghai, China) was added to each well and incubated for 10 min. The cell status was then observed using a laser confocal microscope. For cytotoxicity testing, 100 µL of CCK-8 solution (Beyotime, Shanghai, China) was added to each well, and after 30 min of incubation, the optical density (O.D.) at 540 nm was measured using a microplate reader to evaluate cell viability.

## 3. Results and Discussion

### 3.1. Morphological and Compositional Analysis

**Figure 4:**
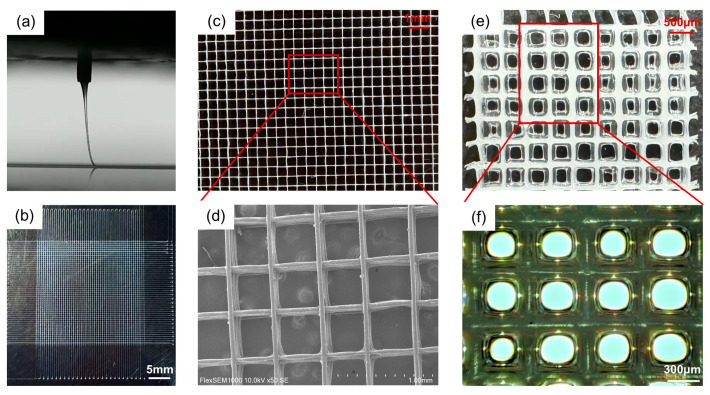
Morphology of scaffolds. (**a**) Jet imaging during the MEW printing process. (**b**–**d**) show the morphology of the pristine PCL fibrous scaffold at different magnifications. (**e**,**f**) The surface morphology of the fiber scaffold after 1 wt% SA hydrogel grafting.

As shown in Figure 4a, PCL fibrous scaffolds were printed under the set parameters at a stable jet state with appropriate jet lag. The prepared PCL fibrous scaffold shows an average fiber diameter of 30 µm, a spacing of 0.5 mm, and a height over 1 mm. Following the steps outlined in Section 2.3.1, the hydrogel was successfully grafted onto the surface of the PCL fibers, as shown in Figure 4e,f. As expected, the hydrogel forms uniformly and exclusively on the surface of the fibers, leaving a pore in each cell.

### 3.2. The Hydrophilic Modification of the PCL Scaffold by NaOH Treatment

**Figure 5:**
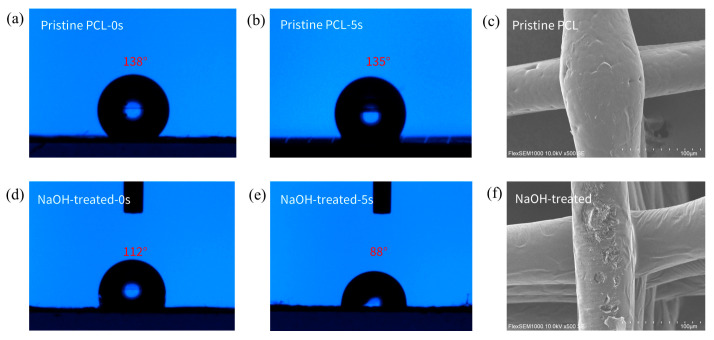
Effects of alkaline modification on wettability and morphology of PCL scaffolds. (**a**) The initial water contact angle (WCA) on the surface of the pristine PCL scaffold. (**b**) The WCA on the surface of the pristine PCL scaffold after 5 s. (**c**) The initial WCA on the surface of the PCL scaffold after NaOH treatment. (**d**) The WCA on the surface of the PCL scaffold with NaOH treatment after 5 s. (**e**,**f**) Changes in the surface morphology of the fibers before and after NaOH treatment.

Figure 5a,b show that the pristine PCL fiber scaffold exhibits poor hydrophilicity, with an initial water contact angle (WCA) of 138°. After 5 s, the WCA decreases to 135°. However, the NaOH treatment significantly improved the hydrophilicity, with the initial WCA reduced to 112° and the WCA after 5 s to 88°. Moreover, the water droplet was completely absorbed by the NaOH-treated scaffold within 10 s. This is attributed to the substitution of -OH groups for hydrogen atoms on the PCL surface, imparting the scaffold with a certain degree of hydrophilicity, which, in later steps of modification, facilitates better hydrogel grafting onto the scaffold surface. Additionally, the NaOH treatment causes etching on the fiber surface, as shown in Figure 5e,f, where noticeable pits can be observed on the fiber surface after NaOH treatment.

### 3.3. Structural Analysis of Composite Scaffolds

**Figure 6:**
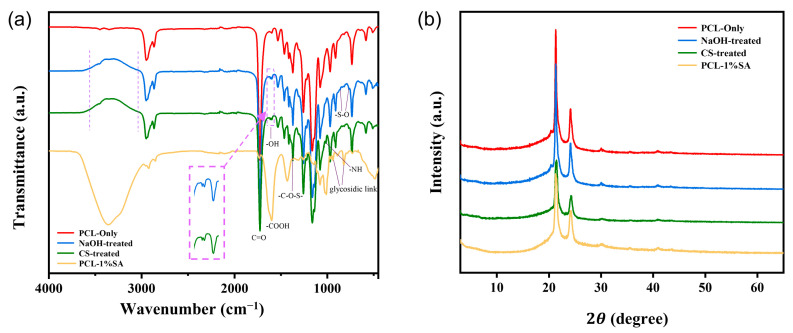
Structure analysis of the scaffold. (**a**) FTIR spectrum of the fiber scaffold. (**b**) XRD spectrum of the fiber scaffold.

As shown in Figure 6a, distinct -OH stretching vibration absorption peaks appear at 3030–3560 cm^−1^ and 1619 cm^−1^, which can be attributed to the hydrophilic modification of the scaffold surface by NaOH treatment and the action of the SDS anionic surfactant. The absorption peaks at 838 cm^−1^ and 765 cm^−1^ correspond to the -S-O group, while the peak at 1370 cm^−1^ corresponds to -C-O-S- groups, all of which are characteristic peaks of SDS. Additionally, an absorption peak at 1016 cm^−1^ resulting from -NH stretching vibrations indicates that CS has been successfully grafted onto the fiber scaffold. For SA, the peaks at 1602 cm^−1^ and 1430 cm^−1^ correspond to the asymmetric and symmetric stretching vibrations of carboxylate groups. The peak at 820–938 cm^−1^ is attributed to the stretching vibration of glycosidic bonds. Additionally, absorption peaks appear at 1083 cm^−1^ and 1021 cm^−1^, corresponding to the stretching vibrations of C-O and C-O-C, respectively.

Figure 6b shows the XRD spectra of PCL and PCL-SA hydrogel composites. Diffraction peaks appear at 2θ = 21.5° and 24.2°, which are attributed to the amorphous structure of the PCL material and alginate. It can be observed that the peaks of the PCL-CS and PCL-CS-SA scaffolds are smaller compared to PCL, which is due to the reduced crystallinity of the material after the PCL scaffold was treated with NaOH and then sonicated in SDS for a period of time.

### 3.4. Pore Size, Thickness, and Porosity Properties of Composite Scaffold

**Figure 7:**
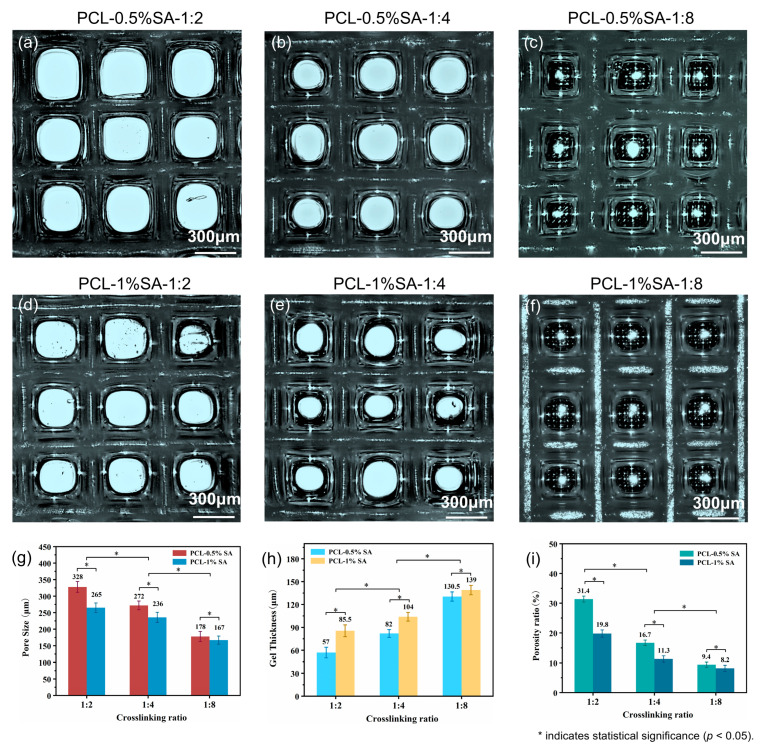
Morphology and structure of the composite scaffolds. (**a**–**c**) Optical microscope images of composite scaffolds at 0.5% SA with different Ca^2+^/SA crosslinking ratios (2:1, 4:1, 8:1). (**d**–**f**) Optical microscope images of composite scaffolds at 1% SA with different crosslinking ratios (2:1, 4:1, 8:1). (**g**–**i**) Pore sizes, hydrogel thickness, and porosity of the different composite scaffolds. *p* < 0.05.

To precisely control the pore features, including pore size, hydrogel thickness, and porosity, the effects of SA concentration and the Ca^2+^/SA crosslinking ratio on pore features are systematically investigated. Specifically, SA concentrations are set at 0.5% and 1%, and the Ca^2+^/SA crosslinking ratios at 2:1, 4:1, and 8:1. Figure 7 shows that increasing the SA concentration or the Ca^2+^/SA crosslinking ratio produces a thicker hydrogel layer on the scaffold. Quantitatively, when the Ca^2+^/SA crosslinking ratio increases from 2:1 to 8:1, the average pore diameter of PCL-0.5%SA scaffolds decreases from 328 μm to 178 μm, and that of PCL-1%SA scaffolds from 265 μm to 167 μm (Figure 7g). At the same time, the hydrogel thickness grows substantially—from 57 μm to 130.5 μm for PCL-0.5%SA and from 85.5 μm to 139 μm for PCL-1%-SA (Figure 7h)—while overall porosity drops from 31.4% to 9.4% and from 19.8% to 8.2%, respectively (Figure 7i). These changes occur because a higher SA content or crosslink density builds a tighter alginate network that fills scaffold voids more completely. It should be noted that by comparing Figure 7g and Figure 7i, as the crosslinking ratio increases, the decrease in the porosity ratio is disproportional with that of the pore size, which is understandable considering the squared relationship between these two parameters. Together, these results confirm that by precisely tuning SA levels and crosslinking, it is possible to tune the pore size, coating thickness, and porosity of the composite scaffolds to suit specific tissue-engineering needs.

### 3.5. Swelling, Degrading, and Mechanical Properties

**Figure 8:**
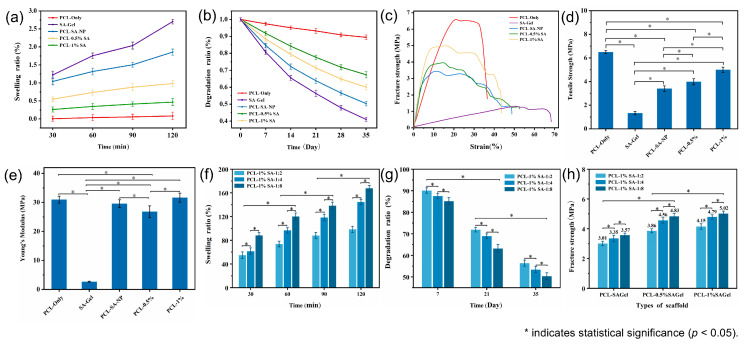
(**a**,**b**) Swelling ratio and in vitro degradation ratio. (**c**–**e**) Stress–strain curves, tensile strength, and Young’s Modulus of the different kinds of scaffolds. (**f**–**h**) Swelling ratio, in vitro degradation ratio, and fracture strength of the composite scaffold under different Ca^2+^/SA crosslinking ratios. *p* < 0.05.

Figure 8 presents the swelling behavior, degradation profiles, and mechanical properties of various scaffold samples. Figure 8a shows the time-dependent swelling ratios of different samples at a Ca^2+^/SA crosslinking ratio of 2:1. The swelling capacity follows the order SA hydrogel > PCL-SA-NP > PCL-1% SA > PCL-0.5% SA > PCL-only, highlighting the dominant role of the hydrogel content in determining the swelling capacity. To further assess the effect of crosslinking, Figure 8d shows that higher Ca^2+^/SA crosslinking ratios lead to increased swelling ratios. This suggests that crosslinking enhances the hydrogel network’s ability to retain water, likely by stabilizing its structure and reducing collapse during swelling.

Figure 8b illustrates the degradation profiles over time, with the degradation ratio ranking identical to the swelling ratio. This consistency stems from the inherently faster degradability of the SA hydrogel compared to PCL. As shown in Figure 8e, increasing the Ca^2+^/SA crosslinking ratio reduces the degradation ratio due to the formation of a denser and more stable hydrogel network with greater mechanical integrity, which is less susceptible to hydrolysis and breakdown.

The stress–strain curves and fracture strengths are shown in Figure 8c–h, respectively. The PCL-only scaffold exhibits the highest tensile strength (6.7 MPa), while the SA hydrogel scaffold displays the lowest (1.2 MPa). Composite scaffolds reinforced with PCL fibers show intermediate strength values. Notably, porous composite scaffolds outperform their nonporous counterparts, likely due to better interfacial adhesion and reduced fiber slippage upon fracture. Additionally, for scaffolds with the same hydrogel concentration, an increase in the Ca^2+^/SA crosslinking ratio leads to higher tensile strength, as stronger crosslinking enhances both internal cohesion within the hydrogel and its interfacial bonding with the fibers. Furthermore, the addition of hydrogel increases the elongation at break, suggesting improved flexibility due to the mobility of polymer chains within the hydrogel matrix.

### 3.6. Biocompatibility of Composite Scaffolds

**Figure 9:**
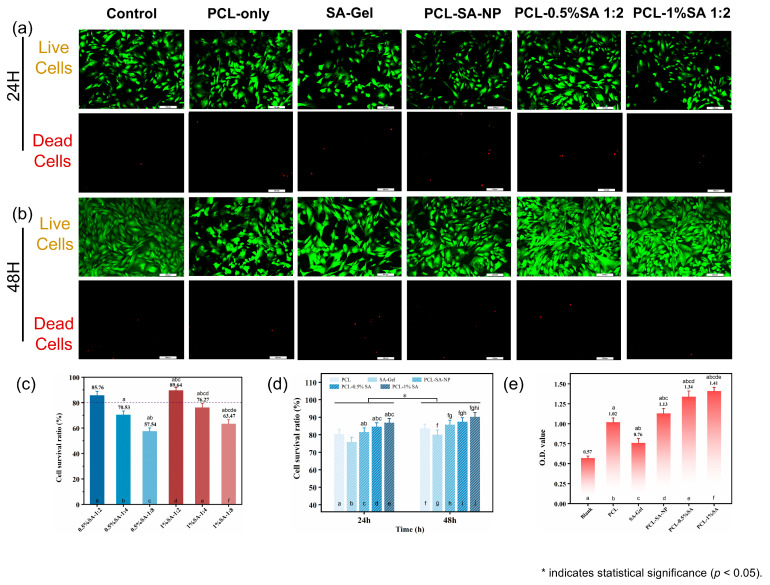
Biocompatibility of the composite scaffolds. (**a**) Live/dead cell staining results for scaffolds after 24 h. (**b**) Live/dead cell staining results for scaffolds after 24 and 48 h. (**c**) Cell viability of composite scaffolds with different pore sizes after 48 h. (**d**) Cell viability of scaffolds after 24 and 48 h. (**e**) Cell adhesion on scaffolds after 48 h. *p* < 0.05. (In Figure 9c–e, the notation ‘a–j’ indicates statistically significant differences when compared with the first through jth columns from left to right).

To evaluate the biocompatibility of different scaffolds, rabbit bone marrow mesenchymal stem cells (BMSCs) were cultured on five types of scaffolds: PCL-only, SA-Gel, PCL-SA-NP, PCL-0.5%SA-2:1, and PCL-1%SA-2:1. Live/dead staining and CCK-8 assays were performed after 24 h and 48 h to assess cell viability and proliferation (Figure 9a–e).

Live/dead staining (Figure 9a,b) revealed that most cells remained viable (green) on all scaffolds after 24 and 48 h of culture, with very few dead cells (red) observed. However, scaffolds incorporating surface-bound SA hydrogel, particularly PCL-1%SA-2:1 and PCL-0.5%SA-2:1, showed visibly higher cell density and more uniform cell distribution compared to the PCL-only or SA-Gel groups. Quantitative analysis of cell survival ratio (Figure 9c) confirmed this trend: PCL-1%SA-2:1 achieved the highest survival ratio at 89.64%, followed closely by PCL-0.5%SA-2:1 (88.76%), while PCL-only and SA-Gel yielded significantly lower viability (57.54% and 70.83%, respectively). These results were further supported by the time-dependent viability data (Figure 9d), in which all SA-containing groups exhibited higher cell survival after both 24 h and 48 h than the PCL-only control.

CCK-8 assay results (Figure 9e) provided additional insight into cell metabolic activity and proliferation. The optical density (O.D.) values at 540 nm were significantly increased in SA-containing composite scaffolds, with PCL-0.5%SA-2:1 and PCL-1%SA-2:1 reaching O.D. values of 1.34 and 1.41, respectively, more than twice that of the PCL-only group (0.76) and three times that of the blank control (0.57). This pronounced enhancement suggests that the hydrogel–fiber composite structure not only supports initial cell adhesion but also promotes sustained cell proliferation.

Mechanistically, the improved cell viability and proliferation on SA-coated PCL scaffolds can be attributed to the synergistic interplay between the structural support of the PCL microfibers and the hydrated, bioactive environment provided by the SA hydrogel layer. The PCL-only scaffold, while structurally robust, presents a relatively hydrophobic and bioinert surface, which may limit initial cell attachment. In contrast, the SA hydrogel coating offers a hydrophilic, ionically crosslinked matrix rich in carboxyl groups, enhancing protein adsorption and facilitating integrin-mediated cell adhesion. Moreover, the optimized Ca^2+^/SA crosslinking ratio (2:1) ensures sufficient hydrogel stability without excessively restricting the mobility of the polymer chains, thus maintaining a soft, compliant interface conducive to cell spreading and proliferation. Interestingly, while the PCL-SA-NP group also showed improved biocompatibility, its nonporous architecture likely limited nutrient diffusion and cell infiltration, leading to slightly lower performance compared to the porous PCL-SA hydrogel coatings.

Overall, these results underscore the potential of surface-engineered PCL-SA composite scaffolds to enhance cell–material interactions, offering a promising platform for soft-tissue-engineering applications.

### 3.7. Comprehensive Evaluation

**Figure 10:**
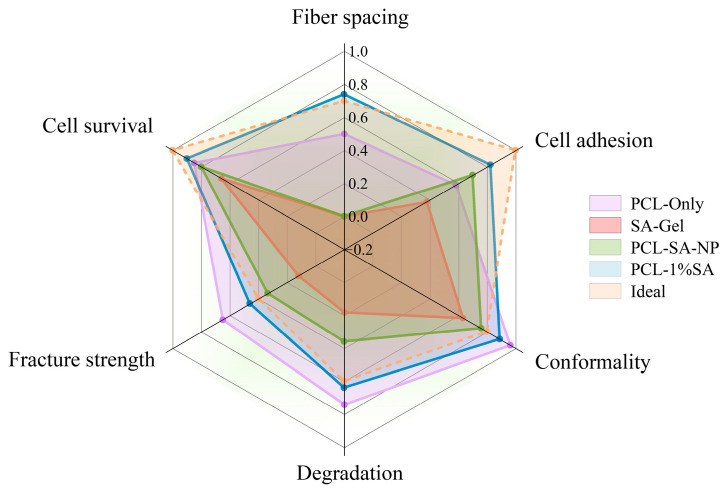
Performance comparison of scaffolds prepared with different approaches.

The performance of samples prepared with different approaches is shown in Figure 10. Among them, ‘Ideal’ represents the suggested performance for scaffolds for cartilage repair based on the literature: the ideal cartilage-tissue-engineering scaffold has pores of 250–300 microns [40], a fracture strain of around 5 MPa [41], a high fidelity ratio, and an appropriate degradation ratio. It can be seen from the figure that the performance of porous composite scaffolds is closest to the ‘Ideal’. Compared with porous composite scaffolds, PCL-only scaffolds have some advantages in mechanical properties, dimensional stability, and degradability, but their biocompatibility and cell adhesion are poor. Except for poor cell compatibility, PCL-SA-NP scaffolds show performances significantly inferior to those of porous composite scaffolds, such as those of their compression modulus, dimensional stability, and degradability. The comprehensive performance of SA-Gel is far inferior to that of the other types of scaffolds.

## 4. Conclusions

This study introduces a novel approach to fabricating porous fiber-reinforced hydrogel scaffolds. Compared to traditional nonporous composite scaffolds, SA hydrogel is grafted exclusively onto the surface of MEW PCL fibers through electrostatic adsorption, while preserving the porous architecture of the composite scaffold. Additionally, by adjusting the concentration of SA and the Ca^2+^/SA crosslinking ratio, the thickness of the hydrogel layer is effectively controlled, enabling the fabrication of composite scaffolds with customizable pore sizes. By comparing the swelling, degradation, mechanical properties, and biocompatibility of different scaffolds, it is found that the porous composite scaffold exhibits performance closest to that of an ideal biological scaffold. Furthermore, MEW PCL fibers and hydrogels form an effective combination, which can significantly enhance the functional value of the composite scaffolds. This study provides an effective strategy to enhance the biological performance of fiber-reinforced hydrogel scaffolds, including their cell adhesion and proliferation.

## Data Availability

The dataset is available on request from the authors.
